# 225 Perceptions of Physical Activity and Structured Exercise in South Carolinian Adult Native Americans

**DOI:** 10.1093/eurpub/ckae114.178

**Published:** 2024-09-26

**Authors:** Maria Felicia Cavallini, David J Dyck

**Affiliations:** Limestone University, Gaffney, South Carolina, United States; University of Guelph, Guelph, Ontario, Canada

## Abstract

Due to the current developments of obesity, diabetes, cardiovascular diseases, alcoholism, hypertension and high suicide rates, the overall health of Indigenous citizens is of considerable concern. Physical activity (PA) plays a key role in overall health and well-being. However, appropriate strategies for PA connection, preferences, motivation, and promotion levels for Natives is lacking and in dire need. The purpose of this study is to examine the adult relationship with PA as well as to investigate the perceived influence of PA not only on physical health but mental, emotional, social, and psychological health among adult Native Americans in South Carolina. Fifteen key informant interviews were conducted with diverse adult Native Americans including four Chiefs. Questions pertaining to beliefs, outlook, preferences, attitude, barriers, gym perception, and motivators towards lifestyle PA versus structured exercise were explored and discussed, as well as PA’s relationship to overall wellness. Qualitative methods with phenomenological underpinnings were used. Using the note transcriptions from the non-taped interviews, we concluded lifestyle physical activity (LPA) was overwhelmingly preferred over structured exercise. Natives found LPA to be an essential part of their culture and way of life. In addition to signifying that the U.S. PA guidelines could be met through LPA, the Native American participants indicated that lack of time was their number one barrier (whether real or psychological), and that being outdoors and engaging in LPA was preferred over the gym environment. Health and feeling good, and being happier afterwards were the main motivators to engaging in LPA. Furthermore, South Carolinian Natives felt that regular participation in LPA would positively affect and directly improve mental, emotional, social, and psychological health. A second more quantitative phase (questionnaire) will follow, with a recruitment of as many Native Americans from South Carlina as possible i.e. 300+. Ultimately, the goal is to develop new and innovative culturally based PA intervention strategies and customized programs for the Native American community through a heritage, health, and cultural center to address the PA and wellness needs of Indigenous people while promoting Native need for community involvement, a wholistic approach to life, and connection to land and the environment.

